# Enhancement of the Laser Transmission Weldability between Polyethylene and Polyoxymethylene by Plasma Surface Treatment

**DOI:** 10.3390/ma11010029

**Published:** 2017-12-26

**Authors:** Huixia Liu, Yingjie Jiang, Wensheng Tan, Xiao Wang

**Affiliations:** 1 School of Mechanical Engineering, Jiangsu University, Zhenjiang 212013, China; 18852868861@163.com (Y.J.); wx@ujs.edu.cn (X.W.); 2 Changzhou Key Laboratory of Large Plastic Parts Intelligence Manufacturing, Changzhou College of Information Technology, Changzhou 213164, China; tws.163@163.com

**Keywords:** laser transmission welding, plasma surface treatment, surface modification, polyethylene, polyoxymethylene

## Abstract

Due to their large compatibility difference, polyethylene (PE) and polyoxymethylene (POM) cannot be welded together by laser transmission welding. In this study, PE and POM are pretreated using plasma that significantly enhances their laser transmission welding strength. To understand the mechanism underlying the laser welding strength enhancement, surface modification is analyzed using contact angle measurements, atomic force microscopy (AFM), optical microscopy, and X-ray photoelectron spectroscopy (XPS). Characterization results show that the plasma surface treatment improves the surface free energy, significantly enhancing the wettability of the materials. The increase in surface roughness and the generation of homogeneous bubbles contribute to the formation of mechanical micro-interlocking. The oxygen-containing groups introduced by the oxygen plasma treatment improve the compatibility of PE and POM, and facilitate the diffusion and entanglement of molecular chains and the formation of van der Waals force.

## 1. Introduction

Given their light weight, excellent physical properties, and low cost, an increasing number of polymers have been used in aerospace, automotive, medical, and other fields [1]. The connection structures of dissimilar polymers display good comprehensive properties. Conventional methods of joining dissimilar polymers are adhesive bonding, mechanical joining, friction stir welding, microwave welding, and hot plate welding, among others [2]. These methods may play an important role in different applications; however, these methods have some obvious defects, such as bad joining quality, low joining efficiency and so on. Compared to the conventional joining methods, laser transmission welding has become one of the most potential connection methods among polymers, polymers with composites, and polymers with metals, and been widely used in the automotive, electronic packaging, and medical equipment industries because of its high efficiency, reliability, speed, and aesthetics [3,4,5,6].

However, in general, due to the poor compatibility and melting point difference, laser transmission welding performance of dissimilar polymers is poor, and even they cannot be welded together. In recent years, improving the laser transmission welding performance between hard-to-weld dissimilar polymers has been the focus of many studies, and scholars from all over the world have attempted to solve this welding problem by various methods. In 2013, Kim et al. [7] studied the laser transmission welding of polypropylene (PP) and polycarbonate (PC), which cannot be welded initially, and they successfully achieved the welding of PP and PC through the grafting modification of PP. In 2015, Liu et al. [8] successfully connected polyethylene (PE) and polyamide 66 (PA66) through the grafting modification of PE. In 2016, Liu et al. [9] coated a thin layer of Al on the glass-fiber-reinforced PA66 surface by using cold spraying technology and successfully achieved the laser lap welding of PA66 and PC. Wang et al. [10] enhanced the laser welding strength of polymethyl methacrylate (PMMA) and polybutylene terephthalate (PBT) by using PC film as the intermediate layer. In 2017, Liu et al. [11] enhanced the laser transmission welding strength of polyvinyl chloride (PVC) and polyamide 66 (PA66) sputtered aluminum thin film through magnetron sputtering. Grafting modification, coating, and adding intermediate layer can enhance the laser transmission welding performance of dissimilar polymers. However, these methods also have some disadvantages, such as changes in the properties of the material itself, the complex process (adding other materials), and high cost. Compared with these modified technologies and methods, low-temperature plasma surface treatment has been widely used in polymer surface modification as an efficient and environmentally-friendly technology. In addition, due to the advantages of rapid response, obvious effect, and unchanged the appearance and bulk properties of the materials, the new surface modification method has become a research hotspot [12,13].

Polyethylene (PE) and polyoxymethylene (POM) are widely used in the automotive, electronic appliance, and machinery industries worldwide [14]. PE is a crystalline and non-polar polymer, whereas POM is a crystalline and weak polar polymer that does not have functional groups in the molecular chains. These properties cause the poor compatibility between PE and POM, and prevent them from being welded by laser transmission welding. To solve this problem, this study proposes the use of oxygen plasma surface treatment for the first time to significantly enhance the welding strength between PE and POM. Furthermore, the mechanism underlying the welding strength enhancement between PE and POM after plasma surface treatment was studied.

## 2. Experimental Section

### 2.1. Experimental Details

Plasma is a kind of approximate neutral aggregate composed of electrons, positive ions, and neutral particles, generated by gas discharge [15]. Plasma treatment is an effective method for the surface modification of polymers [16]. In this study, a plasma treatment apparatus (HD-1B, Changzhou Zhongke Changtsi Plasma Processing Apparatus Plasma Technology Co., Ltd., Changzhou, China) was used. The samples to be treated were placed in the instrument’s reaction chamber made of hard and high temperature resistant glass. For the plasma pretreatment, the output power of 150 W was used with the working gas of oxygen and the pressure of 20 Pa. The pretreatment time was 120 s. The materials after plasma pretreatment were placed for 90 min, and then the laser transmission welding experiments were carried out.

**Figure 1:**
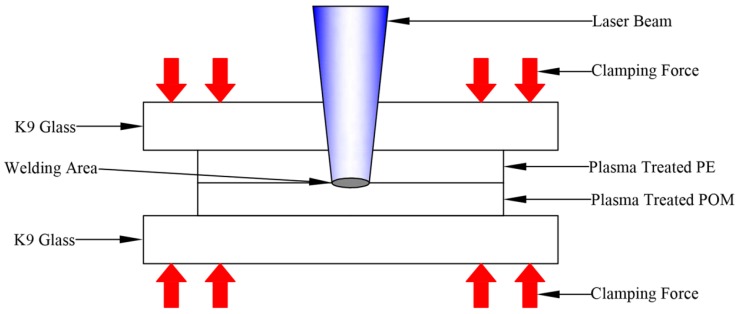
The schematic of laser transmission welding between PE (polyethylene) and POM (polyoxymethylene).

For the laser transmission welding, a Compact 130/140 semiconductor continuous laser made by Dilas was used. The detailed characteristics of the laser source are shown in Table 1. In the experiment, the PE sample after plasma treatment was used as the upper transparent material, and the POM sample after plasma treatment was selected as the lower material. The clearweld coated on the POM surface was used as the laser absorber to absorb the laser energy. The schematic of laser transmission welding between PE and POM after plasma pretreatment is shown in Figure 1. The laser transmission welding experiment was performed in the form of the lap joint, using the K9 glass as the clamping layer, and a certain clamping pressure of 0.5 MPa was applied. The process parameters and limits are shown in Table 2. Three replicates were performed for each test condition.

### 2.2. Materials and Methods

The materials used in this study were PE (60550AG, Lanzhou Petrochemical Company, Lanzhou, China) and POM (Hostaform C9021, Ticona, Florence, KY, USA), and the dimensions of the materials were 50 mm × 20 mm × 2 mm formed by injection molding. The main properties of materials are shown in Table 3. Impurities on the samples were removed with an ultrasonic cleaning machine. Then, the cleaned samples were placed in a drying machine for subsequent plasma surface treatment and laser transmission welding experiment.

**Figure 2:**
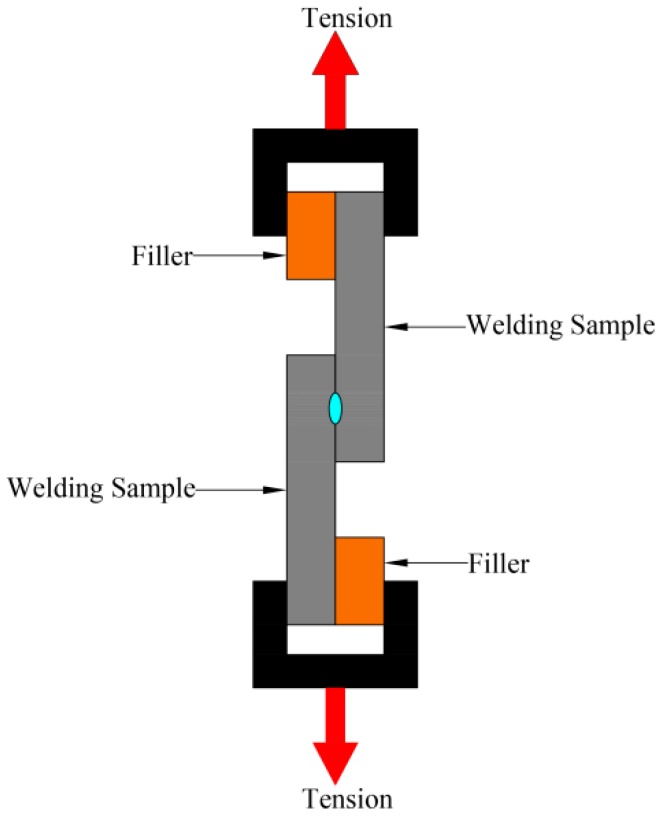
The schematic of the lap shearing test.

The UV–vis–NIR Spectrophotometer (Cary 5000, Varian Company, Milpitas, CA, USA) was used to test the transmissivity of the samples, for which the measured wavelength range was 400–1200 nm. The surface morphology of the samples was characterized by AFM (Dimension Edge, Bruker Nano, Inc., Billerica, MA, USA). Changes in the chemical composition and chemical bond state of the polymer surface before and after plasma treatment were analyzed via XPS. An ultra-depth optical microscopy (VHX-1000, Keyence Corporation, Osaka, Japan) was used to observe the micro morphology of the welding area. After laser welding, the lap shearing test of the joints was performed using the universal tensile machine (UTM4104, Shenzhen Suns Technology Co., Ltd., Shenzhen, China). The lap shearing test finally broke the joints through loading tension at both ends of the welding joints, and the schematic of the lap shearing test is shown in Figure 2. In this paper, the welding strength is measured by shear stress, and the shear stress is calculated as Formula (1)

(1) $$\sigma =\frac{F}{W\times L}$$

where *σ* is the shear stress (MPa), *F* is the maximum tensile force in the tensile test (N), *W* represents the weld seam width (mm), and *L* represents the weld seam length (mm).

Contact angle measurement (OCA40, Dataphysics Instruments GmbH, Filderstadt, Germany) was performed to study the change in contact angles of the polymer surface. The surface free energy of the polymer was calculated using the method of Ownes; this method takes into account the polar components ($\gamma^{P}$) and dispersive components ($\gamma^{D}$) of the surface free energy (γ) and needs to use two test liquids. The relevant formulas are shown below [17]:

(2) $$\gamma =\gamma^{D}+\gamma^{P}$$

(3) $$\gamma_{l1}\left(1+\cos\theta_{1}\right)=2\left[{\left(\gamma_{l1}^{D}\gamma^{D}\right)}^{0.5}+{\left(\gamma_{l1}^{P}\gamma^{P}\right)}^{0.5}\right]$$

(4) $$\gamma_{l2}\left(1+\cos\theta_{2}\right)=2\left[{\left(\gamma_{l2}^{D}\gamma^{D}\right)}^{0.5}+{\left(\gamma_{l2}^{P}\gamma^{P}\right)}^{0.5}\right]$$

where $\gamma_{l1}$ $\gamma_{l2}$ represents the surface tension of the two types of liquid, $\theta_{1}$ $\theta_{2}$ represents the surface contact angle using different test liquids, and $\gamma_{l1}^{D}$ $\gamma_{l1}^{P}$ and $\gamma_{l2}^{D}$ $\gamma_{l2}^{P}$ represent the dispersive and polar components of the surface tension of the different test liquid, respectively.

In this study, pure water and ethylene glycol were used as test liquids for contact angle measurement. The surface tension parameters of pure water were $\gamma_{l1}$ = 75.0 mN/m, $\gamma_{l1}^{D}$ = 21.6 mN/m, and $\gamma_{l1}^{P}$ = 53.4 mN/m, whereas those of ethylene glycol were $\gamma_{l2}$ = 48.0 mN/m, $\gamma_{l2}^{D}$ = 29.0 mN/m, and $\gamma_{l2}^{P}$ = 19.0 mN/m.

## 3. Results and Discussion

### 3.1. Transmissivity Analysis and Lap Shearing Test

**Figure 3:**
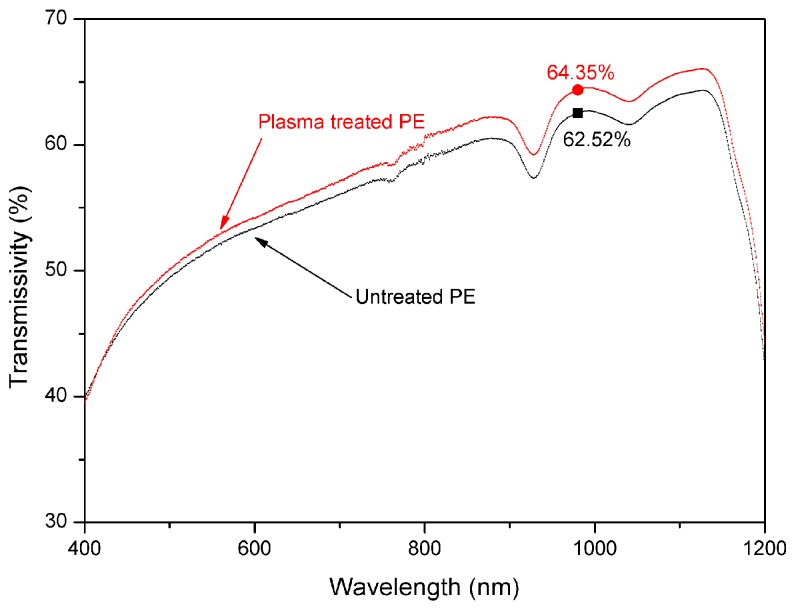
The transmissivity of PE before and after plasma treatment at different wavelengths.

The optical properties of the polymers have a great influence on the welding quality when the polymers are welded by laser. For the upper transparent polymer, the transmissivity of the material is important [18,19]. The output wavelength of the laser used in this study is 980 nm. Figure 3 shows the transmissivity of PE before and after plasma treatment at different wavelengths. The transmissivity of untreated PE is 62.52% when the wavelength is 980 nm. The transmissivity of the plasma-treated PE was increased to 64.35%. PE has high transmissivity and it is suitable to be used as the upper material for the laser transmission welding.

**Figure 4:**
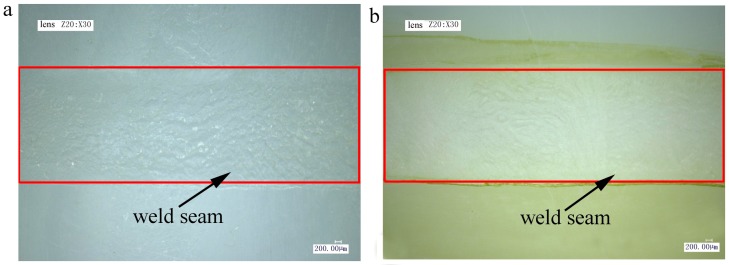
The weld seam of welding samples after shearing test: (**a**) PE; (**b**) POM.

Given their poor compatibility, PE and POM cannot be welded originally by laser transmission welding (almost no connection force). There is no real weld seam between the untreated PE and POM. However, after plasma surface treatment, the plasma-treated PE and POM were successfully welded together. The lap shearing test is an important method to measure the welding strength of welding samples. Measured by the shearing tests, when the laser power is 30 W and the scanning speed is 5 mm/s, the welding strength between the plasma-treated PE and POM is highest about 6.1 MPa. The welding sample of plasma-treated PE and POM after shearing test is shown in Figure 4. The weld seam of the weldment is symmetrical and smooth without obvious defects, such as large bubbles, ablation, and asymmetrical welding seam. It can explain that the plasma surface treatment can improve the welding performance a lot [20]. The mechanism underlying the welding strength enhancement between the plasma-treated PE and POM was investigated below.

### 3.2. Surface Energy Analysis

**Figure 5:**
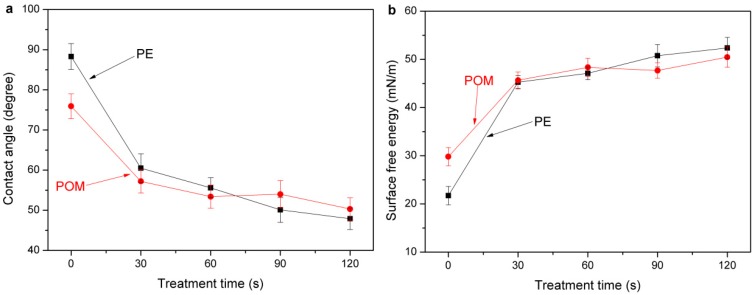
Variation in surface contact angles (**a**) and surface free energy (**b**) of PE and POM.

The contact angles of the PE and POM surfaces are shown in Figure 5a. The surface of the untreated PE is hydrophobic with a water contact angle of 88.3°, whereas that of the untreated POM is slightly hydrophilic with a water contact angle of 75.9°. After plasma treatment, the surface contact angles of PE and POM considerably decrease even with a short plasma treatment time, and the surface hydrophilicity also improves with treatment time.

The changes in the surface free energy of PE and POM after plasma treatment are shown in Figure 5b. The surface free energy of the untreated PE is small (21.7 mN/m) like in other polyolefins. It is noticeable that the surface free energy of the treated PE and POM significantly increases with the plasma treatment time, and the surface free energies of PE and POM are increased to 52.4 mN/m and 50.5 mN/m, respectively. Other scholars reported that the formation of oxygen-containing groups, such as C–O, C=O, and COO, contribute to the enhancement of hydrophilicity and surface free energy. The surface free energy of the materials is improved after plasma treatment, significantly improving the wettability and adhesion of the materials.

### 3.3. Morphology Analysis

**Figure 6:**
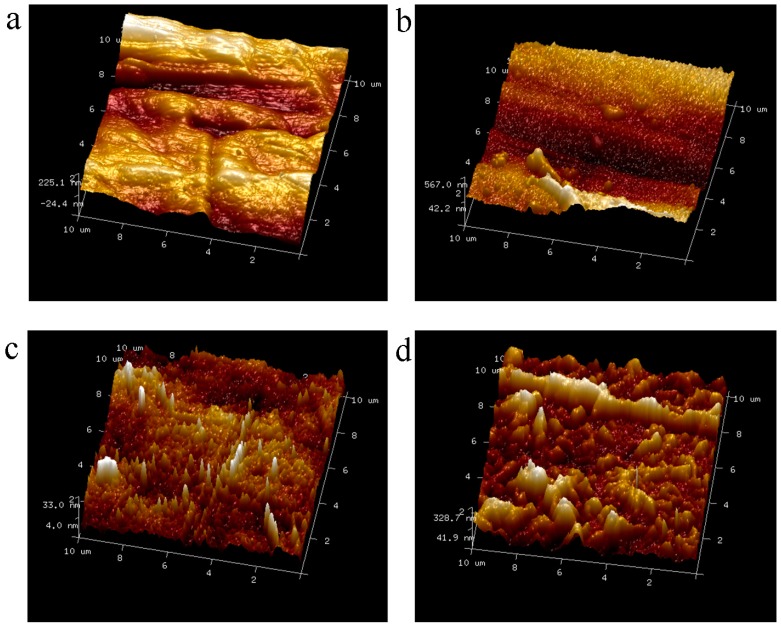
AFM images of polymer surfaces: (**a**) untreated PE; (**b**) treated PE; (**c**) untreated POM; (**d**) treated POM.

**Figure 7:**
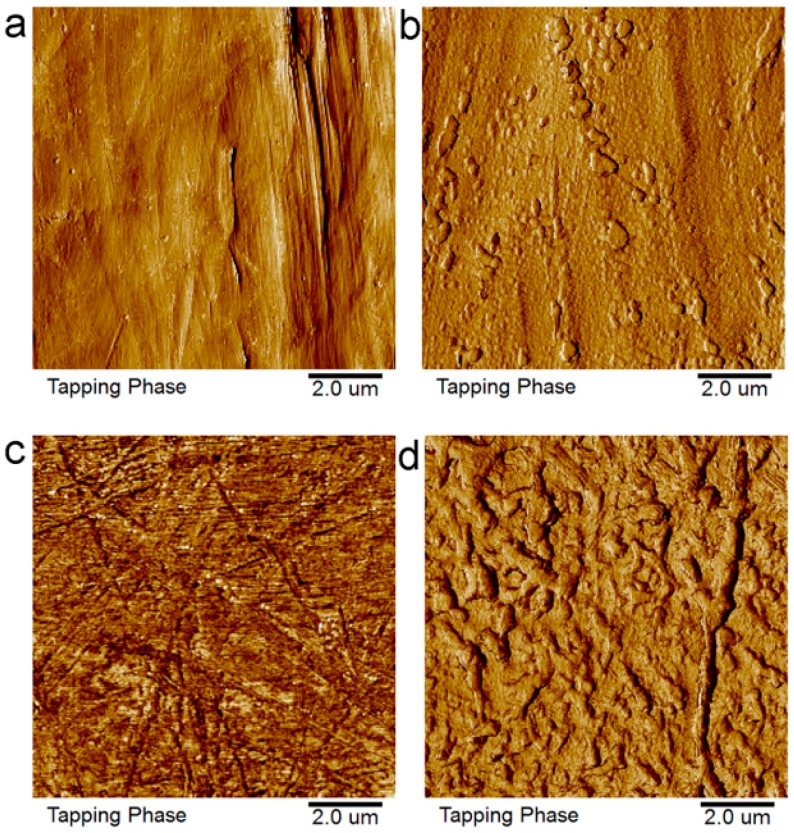
AFM phase images of polymer surfaces: (**a**) untreated PE; (**b**) treated PE; (**c**) untreated POM; (**d**) treated POM.

Figure 6 and Figure 7 show the AFM images of the untreated and treated PE and POM. The surfaces of the untreated PE and POM are relatively smooth. However, the surface morphology of the polymers is changed after plasma treatment, and it is roughed in nanoscale. When the polymer surface is treated with reactive gas plasma, the high-energy particles in the plasma—such as electrons and ions—constantly impinge the polymer surface, and sputter and chemical erosions occur on the polymer surface. The treated surface produces numerous nanoscale hillocks and pits, increasing the surface roughness and specific surface area of the polymers and improving the surface friction coefficient. Furthermore, it can be obviously observed from Figure 7 that the etching effect of the plasma on the POM is better than that of the PE. The increased roughness makes the upper and lower melded materials easy to form mechanical micro-interlocking in the laser welding process. In addition, it increases the contact reaction areas between polymers, easily forming van der Waals force. The increase in surface roughness improves the welding strength between PE and POM. Pandiyaraj et al. [21] indicated that the surface roughness of the plasma-treated polymer is significantly increased, which enhances the mechanical interlocking between the polymers and plays a significant role in the improvement of the polymer bond properties.

**Figure 8:**
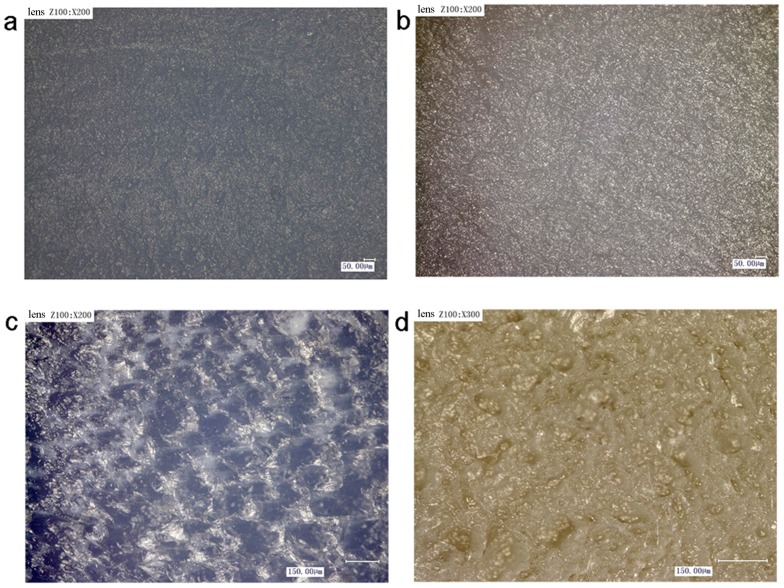
The micro morphology of welding area: (**a**) untreated PE; (**b**) untreated POM; (**c**) treated PE; (**d**) treated POM.

Due to the poor compatibility of PE and POM, the molecular chains cannot spread mutually to form the van der Waals force during laser welding. This leads to the poor welding performance between the untreated PE and POM (almost no connection force), and the micro morphology of the welding area of PE and POM without plasma pretreatment is shown in Figure 8a,b. It can be seen that there are few bubbles in the welding area, and the welding area is smooth. Due to their poor compatibility, the melted polymers heated by laser radiation cannot diffuse mutually during laser welding, and the melted materials are cooled and solidified rapidly after laser scanning.

Figure 8c,d show the micro morphology of the welding area of PE and POM after plasma pretreatment. Compared with the untreated PE and POM, there are a lot of uniform bubbles in the welding area of the treated PE and POM. It shows that the polar groups produced by plasma treatment increase the compatibility of the polymers. It is beneficial to the diffusion and entanglement of molecular chains, consequently producing a certain welding strength in laser welding process. The high pressure generated by these uniform bubbles can force upper fused PE material to the pits and cracks on the surface of lower POM material. In addition, we can see from the Figure 7 that the plasma etching effect of POM is remarkable, and the POM surface produces numerous nanoscale hillocks and pits. Under the double effects of bubbles and etching, the two polymers achieve micro-interlocking mechanism in the laser welding process, increasing the welding strength. Katayama et al. [22] also found that these high-pressure uniform bubbles are advantageous to enhance the welding strength during laser welding.

### 3.4. Chemical Composition Analysis

**Figure 9:**
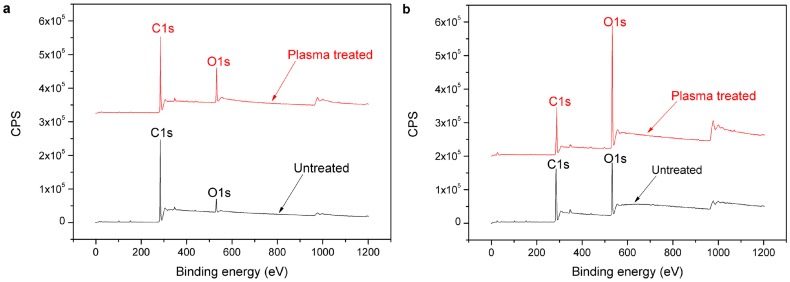
XPS (X-ray photoelectron spectroscopy) spectra of polymer surface: (**a**) PE; (**b**) POM.

XPS analysis was performed to study the changes in chemical composition of polymer surface before and after plasma treatment. Figure 9 shows the XPS spectra of the untreated and treated PE and POM. The surface of the untreated PE contains carbon and little oxygen, however, the content of C1s decreases while the content of O1s remarkably increases after plasma treatment. This phenomenon also occurs on the surface of POM. The content of oxygen on the surface of the treated POM observably increases. The O/C ratio improves from 5.59 at % to 19.08 at % for the PE surface and from 32.97 at % to 77.22 at % for the POM surface (Table 4). These results suggest that the oxygen is incorporated onto the surface of the polymers after plasma treatment. In the process of plasma surface modification, the high-energy particles in the plasma continue to bombard the polymer surface, and then the chemical bonds of the molecules can be opened, generating free radicals. When the polymer is exposed to air or in the presence of oxygen, these free radicals combine with other atoms or molecules, especially oxygen, and a large amount of oxygen-containing groups will be introduced on the surface of polymers, such as –COOH, C–O, C=O, and –OH [23].

**Figure 10:**
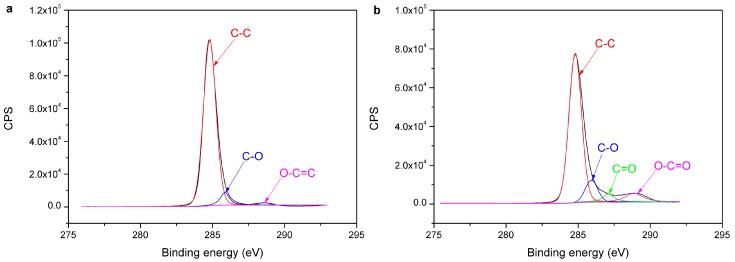
C1s spectra of polymer surface: (**a**) untreated PE; (**b**) treated PE.

**Figure 11:**
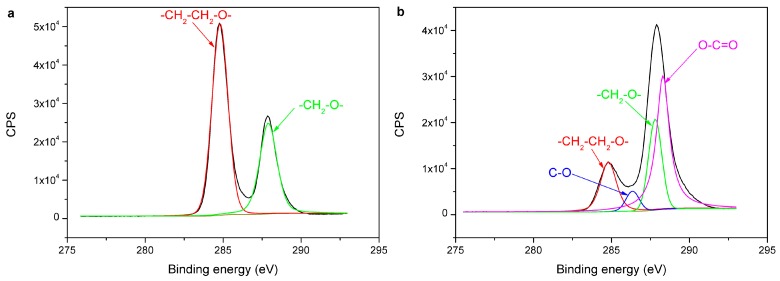
C1s spectra of polymer surface: (**a**) untreated POM; (**b**) treated POM.

Figure 10 shows the C1s spectra of the untreated and treated PE. The C1s spectrum of the untreated PE contains three peaks with the binding energy of 284.80 eV for C–C, 285.95 eV for C–O, and 288.87 eV for O–C=O. The C1s spectrum of the treated PE also contains these three peaks at 284.80 eV, 285.95 eV, and 288.87 eV; furthermore, the new peak at 287.32 eV is shown in the spectra, which may be assigned to C=O [24]. The C1s spectra of the untreated and treated POM are shown in Figure 11. The C1s spectrum of the untreated POM contains two peaks at 284.80 eV and 287.92 eV, which may be attributed to –CH_2_–CH_2_–O– and –CH_2_–O– respectively [25,26]. Aside from these two peaks, two new peaks at 286.35 eV and 288.30 eV are presented in the C1s spectrum of the treated POM, which may be assigned to C–O and O–C=O, respectively [21]. The percent peak area of XPS C1s spectra of PE and POM surfaces before and after plasma treatment are listed in Table 5. There are some oxygen-containing groups produced on the polymer surface after plasma treatment. The type and number of oxygen-containing groups are remarkably increased. The oxygen-containing groups are introduced in the treated PE and POM surfaces, and these groups have a certain polarity, reducing the polarity difference between PE and POM. Furthermore, the surfaces of treaded PE and POM contain the same groups, such as O–C=O and C–O, improving their compatibility. Thus, the molecules are easily diffused and entangled in the laser welding process to form the van der Waals force. These oxygen-containing groups improve the welding strength of PE and POM. Hopmann et al. [27] suggested that in the laser welding of the dissimilar polymers, the polarity of the plasma treated polymers to each other is aligned, so the weldability of the polymers is possible.

## 4. Conclusions

Plasma surface treatment was used to modify PE and POM surfaces. After oxygen plasma treatment, PE and POM, which cannot be welded originally, were successfully welded by laser transmission welding. Plasma surface treatment remarkably improved the surface free energy, improving the wettability of the polymer surfaces. Morphology analysis showed that plasma surface treatment increased the surface roughness of polymers, resulting in numerous nanoscale hillocks and pits on the treated polymer surfaces, especially on the POM surface. Furthermore, compared with the untreated PE and POM, there are a lot of uniform bubbles in the welding area of the treated PE and POM. The high pressure generated by these uniform bubbles can force upper fused PE material to the pits and cracks on the surface of lower POM material. The increase in polymer surface roughness and the generation of homogeneous bubbles allowed the easy formation of mechanical micro-interlocking in the laser welding process. The results of XPS revealed that oxygen is incorporated onto the polymer surfaces after plasma surface treatment. The oxygen-containing groups, such as O–C=O and C–O, improved the compatibility between PE and POM. It facilitated the diffusion and entanglement of molecular chains, caused the formation of the van der Waals force, and enhanced the laser transmission weldability between PE and POM.

## Figures and Tables

**Table 1:** Laser source characteristics.

Characteristics	Value	Unit
Maximum output power	130	W
Output wavelength	980 ± 10	nm
Beam shape	circular	-
Minimum beam diameter	700–800	μm
Fiber core diameter	400	μm
Fiber connector type	SMA 905	-
Numerical apertur	0.22	NA
Operating temperature	15–35	°C
Storage temperature	5–50	°C
Cooling system	air	-

**Table 2:** Process parameters and limits.

Parameter	Limits				
Laser power/(W)	24	26	28	30	32
Laser scanning speed/(mm/s)	3	4	5	6	7

**Table 3:** Main properties of polymers.

Property	PE	POM
Density (g/cm^3^)	0.94	1.39
Specific heat J/(kg·K)	2300	1300
Thermal conductivity (W/mK)	0.44	0.31
Melting temperature, T_m_ (°C)	131	164
Ultimate tensile stress (MPa)	28	58

**Table 4:** Element content of PE and POM before and after plasma treatment.

Polymers	C1s (at %)	O1s (at %)	N1s (at %)	O/C (%)
Untreated PE	94.50	5.28	0.22	5.59
Treated PE	83.11	15.86	1.03	19.08
Untreated POM	74.98	24.72	0.30	32.97
Treated POM	56.14	43.35	0.51	77.22

**Table 5:** Percent peak area of XPS C1s spectra of untreated and treated PE and POM.

	Untreated (at %)	Treated (at %)	Possible Functional Groups
PE			
284.80 eV	92.34	69.79	–C–C–
285.95 eV	6.62	17.06	–C–O
287.32 eV	-	5.06	–C=O
288.87 eV	1.04	8.09	O–C=O
POM			
284.80 eV	64.13	18.69	–CH_2_–CH_2_–O–
286.35 eV	-	5.45	–C–O
287.92 eV	35.87	24.08	–CH_2_–O–
288.30 eV	-	51.78	O–C=O
